# The Practical Medicine Series of Year Books

**Published:** 1907-04

**Authors:** 


**The Practical Medicine Series of Year Books.** Ten volumes. Series of 1906. Issued under the general editorial charge of **Gustavus P. Head,** M.D., Professor of Laryngology and Rhinology in the Chicago Post-Graduate Medical School. Vol. VII. Pediatrics and Orthopedic Surgery, edited by **Drs. Isaac A. Abt** and **John Ridlon.** Chicago: The Year Book Publishers. Series 1906. (Price, $1.25; entire series, $10.00).

Nearly or quite three-fourths of this book is devoted to the topic of pediatrics. This portion is a concise showing of the progress in this branch for the year embraced in the resume of the compilers.

If there is anything in the orthopedic line which has escaped the vigilance of the authors of this excellent number of the year book series it is not worthy of attention. The arrangement of material and its presentation are all that could be desired and it constitutes a perfect resume of the entire field without reservation or particular condemnation. There are of course some things which apparently do not meet the approval of the writers but it adds to the value of the book that these points are not unequivocally condemned.

